# The economy-security conundrum in American grand strategy: foreign economic policy toward China from Obama to Biden

**DOI:** 10.1007/s42533-022-00120-3

**Published:** 2022-11-28

**Authors:** Zeno Leoni

**Affiliations:** Defence Studies Department, Lau China Institute, King’s College London, London, UK

**Keywords:** Grand strategy, US–China relations, Biden foreign policy, Decoupling

## Abstract

This article argues that in a Janus-faced Liberal International Order, American grand strategy faces an economy-security conundrum. Tensions between transnational economic networks and national security concerns require different administrations to square a balance between important yet competing interests. This conundrum is especially challenging for Washington, D.C., when dealing with China. Indeed, while the Chinese and the American economies are interdependent, China continues to rise, with revisionist demands, and outside of the US-led system of alliances. The economy-security conundrum is managed by different presidents who pursue similar long-term objectives but through different approaches, leading to both strategic continuity and policy changes between one administration and the next. Former US President Barack Obama’s Trans-Pacific Partnership, subsequent President Donald Trump’s trade war, and President Joe Biden’s Build Back Better World (B3W)—most recently rebranded as Partnership for Global Infrastructure and Investment (PGII)—are indeed different policies, but all are aimed at either compelling China to abide the free-market rule of law or to separate from the West, especially regarding strategic and future industries.

## Introduction

This article provides an analysis of the economy-security conundrum in US foreign policy toward China. It advances a twofold argument. The economic interdependence of the United States with a geopolitical rival like China requires effort to strike a balance between the economic interests of private companies and the national security interests of the state. This balance is addressed by different presidents and administrations who pursue similar long-term, strategic goals but through different policies: while American grand strategy does not change, one can see different ways in which it is operationalized. Former US President Barack Obama’s Trans-Pacific Partnership, subsequent former President Donald Trump’s trade war, and President Joe Biden’s Build Back Better World (B3W)—most recently rebranded as Partnership for Global Infrastructure and Investment (PGII)—and Indo-Pacific Economic Framework (IPEF) are policies that have sought to implement the same long-term goal—compelling China to abide the free-market rule of law. That said, each administration has pursued this goal with a different approach.

This conclusion is achieved by building on recent neorealist contributions to literature on American grand strategy and the flaws of liberal hegemony identified by Barry Posen (2014), Stephen Walt (2018), and John Mearsheimer (2018). These authors agree that American grand strategy since the end of the Cold War has been overly ambitious as it sought to spread and enforce liberal values worldwide at the expense of US national power and international leadership. This article does not disagree with this view, and the flaws of liberal hegemony must be acknowledged. However, rather than blaming the military overstretch and lavish defense expenditures, this article maintains that the problem with China’s rise from an American perspective has deeper, structural causes. Contrary to what neorealists have argued, however, liberal hegemony has been highly beneficial to the United States for decades, because it has upheld a world order that once provided a political-economic environment in which the US could prosper.^1^ Yet, the American strategy of liberal hegemony tends to cause “blowback[s]” insofar as this strategy requires, encourages, and support other countries—including potential rivals—to develop economically and to actively stimulate and contribute to the global economy by embracing free-market rule of law (Johnson 2000; Leoni 2021, 74; Lee 2020). In the long-term, this strategy can create geopolitical dilemmas by favoring the rise of systemic rivals.^2^ A deeply consequential example is that of China’s economic and geopolitical rise. Beijing’s opening to free-market elements since 1978 has been advocated by US strategy makers in an attempt at building a globalized order, but with the effect of undermining US geopolitical primacy.^3^ This overstretch of US-led globalization has led Washington, D.C., to face its greatest geopolitical challenge to date. China’s integration within the liberal international order (LIO) requires US strategy makers to maintain a delicate balance between economic and national security interests, and this is a challenging task for the world-leading power whose hegemony has been constructed through the promotion of transnational business. Different presidents and administrations seek to achieve a balance between these two dimensions. Presidents pursue similar grand strategic objectives but do so according to their worldview and/or electoral agendas, displaying competing approaches to the operationalization of US grand strategy (Colucci 2018, 139–140).

The rest of this article is organized in four sections. The first defines the economy-security conundrum while engaging with the literature on the LIO. The LIO, indeed, is riddled with tensions between its two structural pillars, such as an aspatial network of flows of capital, goods, people, ideas, and a territorial order made of sovereign states. The second section continues to reflect on the economy-security conundrum but through the lens of American grand strategy. Here, it is discussed how the US, for the sake of economic interests, has contributed to the rise of a geopolitical rival. Furthermore, it is demonstrated how recent US administrations have struggled to find a balance between economic and security interests. The third section contains a comparison of Obama’s, Trump’s, and Biden’s foreign economic policies toward China. In the fourth section, the conclusion is drawn that, while there was discontinuity in the policies used—possibly caused by differences in their worldviews—the ultimate objectives of the three leaders’ strategies were aligned; they all aimed to discipline or isolate China, especially by intervening in supply chains of critical technology and industries (Porter  2018, 11; Waterman and Stokes 2019, 204). The idea of decoupling is the thread that runs through each strategy, although to different extents and in a crescendo from Obama to Biden. Yet, the latter’s policy to contain China faces limits.

## The economy-security conundrum and American grand strategy

The economy-security conundrum concept captures the competing nature of economic and security interests and the challenge of coordinating these in foreign policy. It is particularly useful at a time of deep globalization and rising political systems that are competing with the Western model. While some of the academic literature implicitly acknowledges the existence of such a conundrum, the label “economy-security conundrum” is not used explicitly. The latter represents a more focused interpretation of the concept of “economy-security nexus,” applied as a broad umbrella that covers the realist-liberal dichotomy; this concept is defined as “how well or how poorly states interact with one another in geopolitics and how this is (or is not) affected by their economic relations” (Pempel 2013, 17). However, while the economy-security nexus offers a broad spectrum of possibilities, the rise of China within a Liberal International Order makes the economy-security conundrum highly relevant. This is because the LIO is a Janus-faced order (Layne 2017, 261). In it, the International System of States (ISS) intersects—but does not merge into—a global order whose distinguishing feature is the transnational flow of capital, goods, people and ideas. Spanish sociologist Manuel Castells pointed out that there is a tension between the “space of flows” and “national elites” because the former is “a-spatial” and “escape[s] the socio-political control of nations” while the latter strives to “preserve their social cohesion, develop the set of rules and the cultural codes by which they can understand each other and dominate the others, thus establishing the ‘in’ and ‘out’ boundaries of their cultural/political community” (2009, 446). This tension has been captured by a variety of scholars who appertain to different theoretical and disciplinary camps. Liberal John Ikenberry explained that the LIO “has been built upon the modern states system and evolving frameworks for managing great power relations” (2011, 21). Marxist David Harvey maintained that while ‘[t]he capitalist operates in continuous space and time,… the politician operates in a territorialized space’, and that the ‘relation between these two logics should be seen, therefore, as problematic and often contradictory’’ (2003, 27, 30).

The economy-security conundrum can be further deconstructed. First, while one can artificially separate economic and security interests, they are in fact more interdependent than the concept suggests. Colin Flint explained that the state-network dichotomy is not a simple one, because states—or sections of them—have been “active agents in promoting [or shaping] transnational networks” (Ibid, p. 180; Farrell and Newman 2019). Doing so allows states to have a closer command of transnational flows and to find a more convenient balance with respect to their economy-security conundrum (Flint 2017, 179). Second, the economy-security conundrum is often the product of tensions stemming from the distribution of power within liberal democracies. That said, it also has an external dimension. Security interests of the US, for instance, may clash with economic interests within its network of allies. Finally, while the economy-security conundrum affects the foreign policy of any capitalist democracy, it has greater implications for the hegemon and main beneficiary of the LIO than for other states. Indeed, the conundrum has been highlighted by a variety of scholars writing on American grand strategy. Posen and Ross envisioned four different, competing approaches to US grand strategy, which “are not entirely mutually exclusive” but “contain fundamental disagreements about strategic objectives and priorities” (1996, 50; 2014, 6). William Pfaff described American grand strategy after the Cold War as “[a]n implicit alliance [of] … international liberals, … and unilateralist neo-conservatives” (Pfaff 2001, 221). Similarly, Walter Russell Mead advanced a distinction between Hamiltonian, Wilsonian, Jeffersonian and Jacksonian traditions in US foreign policy, choosing Wilsonianism and Hamiltonianism as the driving principles (2001).^4^ For Gavin, American grand strategy has sought to “contain, open, and inhibit” simultaneously (2015, 12–19). Such a tension is recognized also by critical scholarship in International Political Economy (IPE), history, and geopolitics. Neil Smith maintained that since the twentieth century in American grand strategy there has been a “contradiction between a spaceless and a spatially constituted American globalism” (2003, 7). Perry Anderson defined it as a special pathway to (global) nationalism, “a *complexio oppositorum* of exceptionalism and universalism” (2013, 6).^5^ Similarly, Doug Stokes pointed out that there is in American grand strategy “a dual ‘national’ and ‘transnational’ logic” that works in favor of both American power and global capitalism (2005, 218).^6^

From these accounts of American grand strategists, one can infer two points of both conceptual and historical value. First, the economy-security conundrum is a spectrum, and in different historical periods US administrations will position on different sides of it. During the Cold War, security interests dominated in foreign policy, as compared to the 1990s, when the philosophy of hyperglobalism found many advocates in Washington, D.C. Second, US strategy-makers in the long-term have set the conditions for the US to become entangled in the inner contradictions of its own strategy and, more broadly, of the type of world order that it has designed since the end of WWII. This is especially the case when dealing with a commercial partner that operates through different values and institutions. The challenge for Washington, D.C. is to contain the rise of China while benefiting from its performance as a crucial locomotive of the global economy.

## China’s rise and the US economy-security conundrum

Observing American grand strategy in a post-unipolar era, prominent neorealist scholars advocated for the US to disengage from global commitments. Stephen Walt stated that a grand strategy of liberal hegemony entails that “the United States must remain much more powerful than any other country,” and that “it should use its position of primacy to defend, spread, and deepen liberal values around the world” (2018, 54). Similarly, John Mearsheimer wrote that “liberal hegemony … invariably leads to policies that put a country at odds with nationalism and realism’ (2018, viii).^7^ It is reasonable to sympathize with neorealist arguments. However, they failed to emphasize the structural flaws of American grand strategy. While liberal hegemony has objectively led the US to become the world’s superpower, US efforts to create a global economy have contributed to the rise of potential challengers. This has been the case with regard to former and potential competitors such as Germany, Japan, and above all China, countries which were encourage and even supported to develop technologically by Washington, D.C.

In contrast with Japan and Germany—which remain tied to the US through formal diplomatic agreements—the rise of China has made managing the economy-security conundrum highly challenging, due to the magnitude of the Chinese economy, its depth of integration within the LIO, and the diplomatic-military isolation of the PRC. As China’s military power increased after the Cold War, Washington, D.C. was struggling to keep “the world open enough” for American and global business while “prevent[ing] the rise of any grand challenge” to American power (Harvey 2003, 84). Yet, it has been noted that subsequent US administrations “significantly marginalised [geopolitical interests] in favor of the economic calculus that has come to rule a neoliberalism dominated by US power” (Smith 2005, 187). Although security interests were prioritized during the Cold War, this was not the case in the long run, with respect to US foreign policy toward China. While the short-term objective of the reproachment with Beijing was to isolate the USSR, Washington, D.C.’s long-term objective was to open the Chinese economy to global capitalism (Kissinger 1971; Kissinger 2011, 235, 243; Nixon 1967, 121). After the Cold War, however, neoliberal ideology had free rein and then-President Bill Clinton convinced, with a passionate speech, the US Congress to support China’s membership in the World Trade Organization (Clinton 2000). Hyperglobalist consensus in the US led to a deliberate strategic oversight of China’s rise, as military spending grew by more than 15 times between the late 1990s and the end of the 2010s, while US defense expenditures doubled (The World Bank 2019a, 2019b). The implications of this are that China has become both Washington, D.C.’s most important partner—because it performs as an engine of the world economy—and greatest challenge—due to growing competition in strategic technological sectors and revisionist geopolitical aspirations in the South China Sea. Furthermore, this growth has allowed China to act more assertively since the late 2010s (Shambaugh 2020, 16). Meanwhile, many realized that China does “ultimately prove a much more formidable economic and strategic competitor” than the USSR, thanks to its state-managed capitalism (Shambaugh 2020, 16, Posen 2014, 18).

The rise of China and tensions between economic and security interests have been especially felt in the Indo-Pacific—while not so much in the MENA region, for instance. In 2011 the Obama administration announced that it intended to make the Asia–Pacific a geopolitical priority (Clinton 2011). In particular, this entailed shifting from a 50–50% distribution of naval assets between the Atlantic and the Asia–Pacific to a 40–60% one (Alexander 2012). The Pentagon worried that in the Western Pacific Ocean, within the First Island Chain, military primacy enjoyed by the United States during the Cold War could no longer be taken for granted (Townshend et al. 2019, 9–11). Furthermore, the rise of a middle class of hundreds of millions in the Asia–Pacific has made this part of the world a highly appealing geopolitical region. Yet, the Obama administration did not implement the pivot in a confrontational manner. Some argued that it engaged with China through three different approaches, reflecting the views of three different departments—Treasury, State, and Defence (Luttwak 2012, 213–247).

While the economy-security conundrum continued to affect US strategy-makers, the Trump administration was able to pursue rebalancing economic and security interests—also thanks to a bipartisan anti-China mood in Washington, D.C. (Zhao 2019, 372–373). Meanwhile, Secretary of Defense James Mattis made clear that “[i]nter-state strategic competition, not terrorism, is now the primary concern in U.S. national security” (National Defense Strategy 2018, 1). Against this backdrop, Trump called for an increase in the number of US warships and sought to refine the US “pivot to Asia” by changing its name to Free and Open Indo-Pacific (FOIP), a more ideologically-charged strategy, which also sought to gain the support of India (2017; Medcalf 2019, 68). Although Trump’s rebalancing of the economy-security conundrum in US-China policy was challenged, the president’s actions against China and a spiralling relationship informed President Biden’s agenda. On the one hand, the Biden administration has acknowledged that China is “the only competitor potentially capable of combining its economic, diplomatic, military, and technological power” (The White House 2021, 8). On the other hand, it stated in a foundational speech on American foreign policy that US–China policy should be “competitive when it should be, collaborative when it can be, and adversarial when it must be”, acknowledging that it is not possible to develop a policy that is satisfactory under all aspects (US Department of State 2021). This approach was further articulated by the recognition that allies play a key role and captured by the phrase “invest, align, compete,” three keywords that emphasized the importance of “international coalition building” in Biden’s China policy (US Department of State 2022; Li 2021).

## US foreign economic policy towards China from Obama to Biden

### Obama’s regionalism and the Trans-Pacific partnership

The Obama administration’s foreign economic policy toward China reflected Obama’s commitment to regionalism. The president’s policy manifested elements of pragmatism and selective engagement with the world’s problems, but the worldview was a Wilsonian one. Obama stated that “power is no longer a zero-sum game;” that “[n]o balance of power among nations will hold;” and that the post-WWII “international order … brought about diplomatic cooperation between the world’s major powers” (2009a, 2009b). Considering this, the Obama administration sought to tackle China’s economic power through the Trans-Pacific Partnership, which already existed under former president George W. Bush but evolved into a far-reaching project under Obama (Capling and Ravenhill 2011, 558). Obama’s TPP was a case of leveraging regionalism as a reaction to the slow performance of multilateral mechanisms and to the inability of the American hegemon to shape the agenda in such institutions. It offered a framework for overcoming China’s and other countries’ opposition in the WTO that emerged during the Doha Round—similarly, Obama pursued US interests in Europe through the Transatlantic Trade and Investment Partnership (TTIP) (Dian 2017, 589–592). Its rationale was about strengthening US-led regionalism in Asia–Pacific while engaging in a non-confrontational fashion with China. The TPP required stringent rules on transparency for state-owned companies, an attempt to discipline China’s state capitalism by stimulating internal reform and bringing Chinese companies onto the terrain of free-market competition, where some strategic sectors of American industries thrive. For this reason, regulating state-owned enterprises (SOEs) served the interests of intellectual property (IP) and information technology (IT) companies of Silicon Valley and other US tech hubs. Above all, obstructing SOEs equated to undermining China’s geopolitical power because economic leavers are pivotal to Chinese grand strategy. Obama promised a commitment to: achieve groundbreaking agreements to liberalize … areas where the United States is a global leader in innovation” (NSS 2015, 17). He stated that “it’s important for us to be making the rules in this region, and that’s exactly what TPP does” (Obama 2016). Obama stressed the relationship between China’s geopolitical threat, international standards, and US hegemony when he stated that “if we fail to get the Trans-Pacific Partnership done … then that void will be filled by China … They will make the rules, and those rules will not be to our advantage” (Obama 2015). These words resonated with an equally evident geopolitical vision in an op-ed written by former secretaries John Kerry and Jimmy Carter, in which they wrote that the success of the TPP will have helped the US to support that system that “has served us so well” (Kerry and Carter 2015). With the TPP, the US would have achieved economic security through international standards while encouraging reform in China. Potentially, this would have led to a favorable balance between economic and security interests in the Asia–Pacific.

### Trump’s nationalism and the trade war

While Obama’s worldview depicted an interconnected global system, former president Trump manifested a Hobbesian, disarticulated worldview where “social and political organization are defined in terms of this or that state” (Agnew 2003b, 98). In his UN speech in 2018, Trump stated that “[w]e reject the ideology of globalism, and we embrace the doctrine of patriotism” (Trump 2018b). Because of his emphasis on “pride” and “fear” for “domination and defeat”, scholars labelled Trump a Jacksonian president (Clarke and Ricketts 2017). This worldview stemmed from a foreign economic policy in contrast to that of the Obama administration, although the long-term goals were the same. While withdrawing American participation from the TPP allowed Trump to respect promises made during the electoral campaign, US opposition to China’s state-led capitalism, cheap export, growing competition in strategic sectors, and intellectual property theft continued in a more pressing, confrontational, and bilateral manner with the use of trade tariffs. Like Obama, Trump stated that the “United States must marshal the will and capabilities to compete and prevent unfavourable shifts in the Indo-Pacific” (NSS 2017, 45). During his Asian tour, Trump denounced the fact that “other countries used government-run industrial planning and state-owned enterprises” engaged in “predatory industrial practices” that could harm American private enterprises and innovation (Trump 2017). The Silicon Valley system—including other American technological hubs—and the increasingly important infrastructural value of the Internet in the global economy, were not only central to Obama’s TPP, but also remained a concern for the Trump administration. US Trade Representative Robert Lighthizer, in fact, stated that there is an “unprecedented threat” posed by China, because “[t]echnology and innovation are America’s greatest economic assets … we must … protect American competitiveness” (Office of the US Trade Representative 2018). Indeed, this highlights once again the importance of international standards for American hegemony and how this depends on a certain relationship between economic and geopolitical interests. However, contrary to Obama, Trump operationalized American grand strategy with a nationalist policy. At the end of May 2018, Trump announced an additional 25% duties for a list of products that included about 1102 separate U.S. tariff lines (ibid. 2018) for a total of $50 billion in 2018 trade value. The logic of this list was to hit products from industrial sectors related to the “Made in China 2025” industrial policy, particularly those regarding “aerospace, information and communications technology, robotics, industrial machinery, new materials, and automobiles” (ibid.). Trade sanctions were published in the Federal Register of June 20, 2018 and became effective July 6 (Federal Register 2018, 28710–28756). On June 18, 2018, the US announced more tariffs worth £200 billion, should have China retaliated against the first round of tariffs (Trump 2018a). These were added to tariffs on US$250 billion worth of Chinese products. Yet, while Trump was determined to mitigate the consequences of the economy-security conundrum, the effectiveness of tariffs was limited by its bilateralism amidst a world economy that moves along multilateral networks—the negotiation between Washington, D.C. and Beijing never reached Phase 2. The problem was addressed by the Biden administration.

### From the B3W to IPEF: Biden’s strategy of positive decoupling

Contrary to recent presidents like George W. Bush, Obama, and Trump, whose worldviews were clearcut, President Biden has not yet left an ideological legacy in US foreign policy. Nonetheless, it is evident that two structural factors have influenced his approach. First, in contrast to his two predecessors, Biden has been a member of the political establishment throughout his career; his foreign policy has not derailed from the pillars of American grand strategy—in the way Trump’s policy did. But, Biden inherited from Obama and Trump the strategy of “pivot to Asia” and a domestic environment increasingly sceptical of China. Considering this, his foreign economic policy toward China has sought to tackle the economy-security conundrum with features that resembled both Obama’s and Trump’s approaches. Like in Obama’s and Trump’s foreign policy, the underlying theme of Biden’s policy has been one of growing dissatisfaction with the LIO, and an effort to reform it in a more US-friendly way. While Biden’s strategy remains undeveloped, the primary theme is pursuing geo-economic re-engineering of the LIO by leveraging US alliances. From an operational viewpoint, Biden has sought to bridge the gap between supply chains and geopolitical objectives; that is, to make trading networks more resilient *vis-à-vis* diplomatic tensions and disruptions. This objective has been set out in the executive order titled America’s Supply Chains, which demanded a review of US supply chains resilience (Biden 2021). The report, submitted by Jake Sullivan and Brian Deese—assistants to the president for National Security and Economic Policy, respectively—stated that “we must reduce our dependence on China and other geopolitical competitors for key products”, adding that a “friend-shoring” that keeps supply chains among allies should be pursued (2022, 7). Two initiatives have stemmed from this geoeconomic necessity: the Build Back Better World (B3W) and the Indo-Pacific Economic Framework (IPEF). If the White House’s fact sheet emphasized that the B3W focuses on “climate, health and health security, digital technology, and gender equity and equality,” the Carbis Bay G7 Summit Communiqué aimed at obtaining greater political alignment around US-sponsored values and standards of the rules-based international order (2021). This was congruent with the advice of Congress, which stated that the Biden administration should “initiate an agenda with G7 and G20 countries on matters relevant to economic and democratic freedoms” on “international infrastructure standards,” “security of 5G telecommunications,” “[a]nti-competitive behavior,” “[p]redatory international sovereign lending,” “[i]nternational influence campaigns” among other things (S.1260—117th Congress 2021-2022, 857–858). This policy represents an attempt at creating a new sphere of influence or economic-technological line of defense, a more exclusive club than the post-WWII LIO which seeks to hinder China from accessing parts of the global economy, including next-generation industries and technologies. Its final goal is to build a LIO 2.0. This policy has been described by the Henry Jackson Society as “positive decoupling” (Rogers et al. 2020). This concept means that the US and some of its partners “may not be able to regenerate self-sufficiency across all strategic sectors” and should accept limited dependency on China in some strategic areas (ibid., 34). However, with projects like the B3W the US and its partners commit to “forcing breakthroughs in frontier technologies that China does not yet dominate, rather than chasing after China’s production of existing products” (ibid.). During the G7 held in Bavaria (Germany), the B3W was rebranded the Partnership for Global Infrastructure and Investment (PGII), although this represents “a continuation and expansion” of the B3W, which seeks to “catalyze international infrastructure financing and development” (Rahman and Ahmad 2022; The White House 2022a).

A second important move, in keeping with the overarching logic of the B3W, was the launch of IPEF, Biden’s attempt at replacing the defunct TPP—which currently the US does not intend to re-join (Barns-Graham 2021). This is a geoeconomic strategy rather than a free trade negotiation—with the specific intent of eroding China’s growing influence in the Indo-Pacific. It provides the US with an opportunity to develop a non-traditional free trade agreement (FTA), as countries joining IPEF will not need to lower their custom barriers. This approach seems to consider the more consensus-based procedures used within ASEAN and the diversity of the Asia–Pacific. Concretely, IPEF is seeking to uphold a US-friendly regional and international order by promoting cooperation to make the world economy digital, resilient, cleaner, and fairer (The White House 2022b).

## The limits to decoupling

Biden’s economic policy, aimed at keeping China out of a US-led sphere of influence, represents a development in the commitment to decoupling within US policy circles. That said, for the GPII and IPEF, the road is not without obstacles. Regarding the GPII, at Carbis Bay G7, the communiqué confirmed that members “will cooperate to address the challenge posed by China” but only “where it is in our mutual interest” (Carbis Bay G7 Summit Communiqué 2021, 19). Furthermore, it was reported that while the US was keen to make most of the G7 and of the B3W about China, Britain and other European countries preferred to identify the G7 spirit with a post-COVID-19 reconstruction of the economy; these countries were sceptical of the US’s hawkish stance towards China (Leoni 2022; Parker and Cameron-Chileshe 2021). Others, like South Korea—invited as a guest—have continued to apply “strategic ambiguity” between the US and China. Nonetheless, the drama of the war in Ukraine and China’s pro-Russia stance might have played in favor of the Biden administration. Due to the fear of a Russian invasion of Europe, US partners have become more lenient toward Washington, D.C.’s demands—at least in the short term. Yet, the concrete operationalization of the GPII remains nebulous and faces several uncertainties, from its reliance on private funding to the political resolve in a broad coalition. IPEF faces challenges that are substantially not different from those of the GPII. Even if IPEF was well received—on the day of the launch, Indo-Pacific countries joined—this framework remains a lame duck. Indeed, the new initiative is causing a degree of “unease” among those regional members that would like to gain access to the American domestic market and take advantage of their competitive production costs (Harris 2022). But for this to happen the US would have to join the Comprehensive Progressive Agreement for Trans-Pacific Partnership (CPTPP). Furthermore, because IPEF is not an FTA, the next president might easily scrap IPEF with an executive order. This flaw, in particular, increases the political charm of China, which could appear as a more politically stable and reliable partner. Meanwhile, this puts the fragility of US grand strategy under the spotlight, with domestic institutions incapable of taking an economic burden for the sake of geopolitical objectives. Overall, it remains unclear what the B3W and IPEF bring to the table, both in terms of resources and opportunities for American allies. In fact, while these frameworks might offer a solution to the economy-security conundrum, US partners will have to be persuaded that they will be able to compensate for the loss of income that any decoupling from China might involve.

Nonetheless, these two frameworks are informed by strategic thinking and represent a sophisticated evolution in US foreign economic policy toward China. More to the point, the comparison between Obama’s, Trump’s, and Biden’s foreign economic policy toward China, provides evidence about two dynamics central to American grand strategy in the post-2008 era. First, tensions between economic and security interests have great influence on US economic policy toward China. In a liberal order made of an international system of states and a spaceless economy, the foreign policy of the hegemon will be in tension with developing a coherent policy toward one of the locomotives of the global economy, namely China. Second, in hindsight and following from the previous point, the trend of US foreign economic policy toward China has been and will continue to be one of decoupling. To balance the economy-security conundrum, it appears logical to assume that this decoupling will be highly selective and is likely to affect only some strategic industries—as in the case of semiconductors—and, above all, next-generation technology, and the green economy.

## Conclusion

This article sought to advance a contribution to the debate on US foreign policy after 2008 and US–China relations by unpacking the tension between national and global interests endemic to US grand strategy. This intervention was necessary to bring fresh insights into the debate stimulated by arguments laid out by neo-realist scholarship. Neorealists such as Posen, Walt, and Mearsheimer pointed out that American grand strategy, especially since the end of the Cold War, has been flawed by its constant attempt at engineering a liberal international order based on international rule of law, liberal democracy, and human rights, among several other principles. They correctly maintained that enforcing such order has undermined American national interests and power. This article, indeed, agreed that the American grand strategy of liberal hegemony has drained the relative power of the hegemon. Yet, it maintained that the main structural flaw of a strategy of liberal hegemony is the intentional integration into the LIO of countries that can become geopolitical rivals of the United States. While American grand strategy represents a synthesis of two self-reinforcing but also contradictory elements—nationalism and globalism—this strategy has contributed to causing the rise of economic competitors, such as Japan and Germany, and systemic rivals, such as China. Managing the economy-security conundrum in a post-American order, furthermore, has become an increasingly challenging task, especially when confronting China. The latter’s strategic interlocking between political power and key—state-owned—industries allows China to leverage on whole-of-government cooperation for the sake of geopolitical objectives. The latest developments in American foreign policy, from the B3W and PGII to IPEF and, more broadly, to calls for decoupling and whole-of-government approaches against security challenges, are a testament to the fact that the US, like other Western governments, is taking a page from China’s book. Cooperation between government and industries, and between close allies, is a key element for facing a competitor like China that can pull different levers of power at once.

## Data Availability

Data were access through secondary sources available online as open source or through library login details.

## Footnotes

1 The neorealist critique focuses on the post-Cold War era. However, this article’s author does not emphasize this watershed to the extent the US after the Cold War ‘adapted its long-standing post-World War II grand strategy to a new era of American dominance’ (Brands 2018, 2).

2 See Lascurettes on ‘ordering-to-exclude’ (2020, 35).

3 Such perspective also allows to provide a more analytical explanation of why the US is on a ‘collision course’ with China (Layne 2017).

4 See also Martel (2015, 361).

5 See also Agnew (2003a, 877–880).

6 Colucci explores additional literature along the realist-idealist divide (2018, 139–140). Departing from the literature so far surveyed, Dombrowski and Reich argued that American grand strategy does not exist because ‘the constraints imposed by diverse operational demands’ lead to ‘a significant gap between … rhetoric and behaviour’, or between strategy and operations (Dombrowski and Reich 2017, 1014–1015).

7 See also Posen (2014, xi).
